# Individual variations and sex differences in hemodynamics with percutaneous arterial oxygen saturation (SpO_2_) in young Andean highlanders in Bolivia

**DOI:** 10.1186/s40101-020-00240-y

**Published:** 2020-10-07

**Authors:** Takayuki Nishimura, Juan Ugarte, Mayumi Ohnishi, Mika Nishihara, Guillermo Alvarez, Yoshiki Yasukochi, Hideki Fukuda, Kazuhiko Arima, Shigeki Watanuki, Victor Mendoza, Kiyoshi Aoyagi

**Affiliations:** 1 Department of Public Health, Nagasaki University Graduate School of Biomedical Sciences, 1-12-4 Sakamoto, Nagasaki, 852-8523 Japan; 2 Department of Human Science, Kyushu University, 4-9-1 Shiobaru, Minami-ku, Fukuoka, 815-8540 Japan; 3 Faculty of Dentistry, Universidad Mayor de San Andres, Av. Saavedra 2244, La Paz, Bolivia; 4 Department of Health Sciences, Nagasaki University Graduate School of Biomedical Sciences, 1-12-4 Sakamoto, Nagasaki, 852-8523 Japan; 5 Department of Human Functional Genomics, Advanced Science Research Promotion Center, Organization for the Promotion of Regional Innovation, Mie University, 1577 Kurima-machiya, Tsu, Mie 514-8507 Japan; 6 National Institute of Public Health, 2-3-6 Minami, Wako, Saitama 351-0197 Japan

**Keywords:** High altitude adaptation, SpO_2_, Physiological variation, Sex difference, Andean highlanders

## Abstract

**Background:**

Many studies have reported specific adaptations to high altitude, but few studies have focused on physiological variations in high-altitude adaptation in Andean highlanders. This study aimed to investigate the relationships between SpO_2_ and related factors, including individual variations and sex differences, in Andean highlanders.

**Methods:**

The participants were community-dwelling people in La Paz, Bolivia, aged 20 years and over (age range 20–34 years). A total of 50 men and 50 women participated in this study. Height, weight, SpO_2_, hemoglobin concentration, finger temperature, heart rate, and blood pressure were measured. Information about lifestyle was also obtained by interview.

**Results:**

There were individual variations of SpO_2_ both in men (mean 89.9%, range 84.0–95.0%) and women (mean 91.0%, range 84.0–96.0%). On Student’s *t* test, men had significantly lower heart rate (*p* = 0.046) and SpO_2_ (*p* = 0.030) than women. On the other hand, men had significantly higher SBP (*p* < 0.001), hemoglobin (*p* < 0.001), and finger temperature (*p* = 0.004). In men, multiple stepwise regression analysis showed that a higher SpO_2_ was correlated with a lower heart rate (*β* = − 0.089, *p* = 0.007) and a higher finger temperature (*β* = 0.308, *p* = 0.030) (*r*^2^ for model = 0.18). In women, a higher SpO_2_ was significantly correlated with a higher finger temperature (*β* = 0.391, *p* = 0.015) (*r*^2^ for model = 0.12). A higher SpO_2_ was related to a higher finger temperature (*β* = 0.286, *p* = 0.014) and a lower heart rate (*β* = − 0.052, *p* = 0.029) in all participants (*r*^2^ for model = 0.21). Residual analysis showed that individual SpO_2_ values were randomly plotted.

**Conclusion:**

Random plots of SpO_2_ on residual analysis indicated that these variations were random error, such as biological variation. A higher SpO_2_ was related to a lower heart rate and finger temperature in men, but a higher SpO_2_ was related to finger temperature in women. These results suggest that there are individual variations and sex differences in the hemodynamic responses of high-altitude adaptation in Andean highlanders.

## Background

Studies of adaptation to high altitude started over 100 years ago, and they focused on increased hemoglobin (Hb) concentrations in Andean highlanders or sojourners in high-mountain areas [1, 2]. This Hb increase is the typical physiological response of humans at high altitude [2–4]. Hb carries less oxygen with increasing altitude because the partial pressure of oxygen in the lung is decreased, and there is decreased oxygen available for diffusion into the blood [5]. This percentage of arterial Hb with oxygen is evaluated by measuring percutaneous arterial oxygen saturation (SpO_2_), and SpO_2_ indicates the oxygen level in the body. A lower SpO_2_ occurs in acute mountain sickness, which can include various severe symptoms [6, 7]. Therefore, to maintain oxygen levels, people who stay at high altitude and Andean highlanders have increased Hb concentrations or SpO_2_.

Although early studies focused on the Andean type of physiological adaptation, such as high Hb and slightly lower SpO_2_ [5, 8, 9], recent studies reported population differences in high-altitude adaptation and suggested that there are different physiological responses to hypobaric hypoxia despite similar levels of hypoxic stress. Interestingly, Tibetan highlanders have lower Hb and SpO_2_ levels than Andean highlanders [10–12].

Previous studies also reported physiological variations and sex differences in highlanders. In Andean highlanders, Beall et al. [5] reported that SpO_2_ was higher in men than in women. Furthermore, higher SpO_2_ was associated with higher Hb concentrations in Ethiopian women [13]. In Tibetans of Nepal, lower SpO_2_ was associated with lower glycosylated hemoglobin (HbA1c) [14]. In other studies, SpO_2_ was an important index for acute mountain sickness (AMS) in Andean highlanders [8], and there were individual variations of SpO_2_ in lowlanders exposed to hypobaric hypoxia [15]. These results suggest that there are individual variations and sex differences in high-altitude adaptation, and SpO_2_ is an important physiological index for evaluating adaptability to high altitude.

Although some studies have reported the Tibetan type of high-altitude adaptation in recent years [1, 4, 14, 16], studies of the Andean type are limited; in particular, few studies have focused on physiological variations from the perspective of physiological anthropology [5, 8]. Therefore, this study aimed to investigate the relationships between SpO_2_ and related factors, including individual variations and sex differences, in Andean highlanders.

## Methods

The participants were community-dwelling people aged 20 years and over (20–34 years old) in La Paz, Bolivia (altitude 3700–4000 m), who were invited to participate in this study in 2016. All participants were born and raised around La Paz. A total of 50 men and 50 women (university students) participated in this study. All participants gave their written, informed consent before the examination. This study was approved by the Ethics Committee of Nagasaki University Graduate School of Biomedical Sciences (No. 16072995).

After an explanation of the experiment and obtaining written, informed consent (30 min), each participant’s height, weight, SpO_2_, hemoglobin concentration, finger temperature, heart rate, and blood pressure were measured. All parameters were measured at a room temperature of 23 to 25 °C, participants stayed in the room for up to 2 h, and they wore normal clothes (not controlled).

SpO_2_ was measured by a finger pulse oximeter (Masimo Radical V 5.0, Masimo Corp., Irvine, CA, USA). Hb concentration and finger temperature on the inner surface of the index finger were measured by an ASTRIM FIT health monitoring analyzer (Sysmex, Kobe, Japan). After measuring SpO_2_, Hb, and finger temperature at the right hand, systolic blood pressure (SBP), diastolic blood pressure (DBP), and heart rate were measured in the left arm in the resting condition by a digital automatic blood pressure monitor (OMRON Model, HEM-7210, Kyoto, Japan). These physiological measurements were measured within 1 h of entering the room. Height (m) and weight (kg) were measured with light clothing and without shoes, and the body mass index (BMI) was calculated as weight/height squared (kg/m^2^). Keeping vertical at each point, height was measured to the nearest 0.1 cm from the top of the head to the heel using a tape measure (0 to 200 cm) attached to the wall. Information about physical activity (walking or doing any equivalent amount of exercise activity more than 30 min a day (yes/no)), current smoking (having one or more cigarettes per day (yes/no)), and alcohol drinking (some alcohol consumption one or more days per week (yes/no)) was obtained by interview. However, physical activity, smoking, and alcohol drinking were not controlled before the measurements.

### Statistical analysis

Variables are presented as means with SD. Student’s *t* test and Fisher’s exact test were used for comparisons between men and women. Pearson’s correlation analysis was used to assess correlations between SpO_2_ and other parameters for all participants, for men, and for women. Multiple stepwise regression analysis was used to assess correlations between SpO_2_ and related parameters (sex, height, weight, BMI, hemoglobin, heart rate, SBP, DBP, finger temperature, current smoking, alcohol drinking, and physical activity) for all participants, for men, and women. To assess the individual variations of SpO_2_, residual analysis was performed using a multiple stepwise regression model in both sexes. The estimated values of SpO_2_ were plotted on the *X*-axis, and the residuals between the measured and estimated values of SpO_2_ were plotted on the *Y*-axis. The 95% confidence interval (CI) of the residuals was calculated as the limits of agreement.

A *p* value of less than 0.05 was considered significant. The data were analyzed using the Statistical Analysis System software package version 9.4 (SAS Institute, Cary, NC, USA).

## Results

There were individual variations of SpO_2_ in both men (mean 89.9%, range 84.0–95.0%) and women (mean 91.0%, range 84.0–96.0%). Table 1 summarizes the characteristics of the 100 participants. Men had significantly higher height and weight than women, but there was no significant difference in BMI. On physiological measurements, men had significantly lower heart rate and SpO_2_ than women. On the other hand, men had significantly higher SBP, hemoglobin, and finger temperature. In other factors, men had significantly higher rates of alcohol drinking and physical activity.

**Table 1:** Characteristics of the study population

	Total (*n* = 100)	Men (*n* = 50)	Women (*n* = 50)	*P* value
	Mean (95% CI)			
Age (years)	25 (24.3–25.4)	25 (24.6–26.0)	24 (23.6–25.2)	0.107
Height (cm)	159.5 (157.8–161.1)	165.5 (164.0–167.0)	153.4 (151.7 -155.1)	< 0.001
Weight (kg)	61.9 (59.9–64.0)	67.7 (64.9–70.5)	56.2 (54.0–58.3)	< 0.001
BMI (kg/m^2^)	24.3 (23.6–25.0)	24.7 (23.7–25.7)	23.9 (22.9–25.0)	0.267
Heart rate (bpm)	77 (74.6–78.9)	75 (71.6–77.5)	79 (75.8–82.0)	0.046
SBP (mmHg)	114 (111.2–116.1)	118 (113.9–121.6)	110 (106.8–112.2)	< 0.001
DBP (mmHg)	66 (63.9–67.3)	66 (63.0–68.4)	66 (63.4–67.6)	0.917
SpO_2_ (%)	91 (90.0–91.0)	90 (89.2–90.6)	91 (90.3–91.8)	0.030
Hemoglobin (g/dl)	14.4 (14.1–14.6)	15.4 (15.2–15.6)	13.3 (13.0–13.6)	< 0.001
Finger temperature (°C)	32.8 (32.3–33.2)	33.4 (32.8–34.1)	32.1 (31.4–32.7)	0.004
	% (95% CI)			
Current smoking (yes)	3.0 (0.0–6.3)	2.0 (0.0–5.9)	4.0 (0.0–9.4)	0.379^a^
Alcohol drinking (yes)	18.0 (10.5–25.5)	26.0 (13.8–38.2)	10.0 (1.7–18.3)	0.037^a^
Physical activity (yes)	48.0 (38.2–57.8)	62.0 (48.6–75.4)	34.0 (20.9–47.1)	0.008^a^

Student’s *t* test for continuous variables

*SD* standard deviation, *CI* confidence intervals, *BMI* body mass index, *SBP* systolic blood pressure, *DBP* diastolic blood pressure, *SpO*_*2*_ saturation of percutaneous oxygen

^a^Fisher’s exact test

**Fig. 1:**
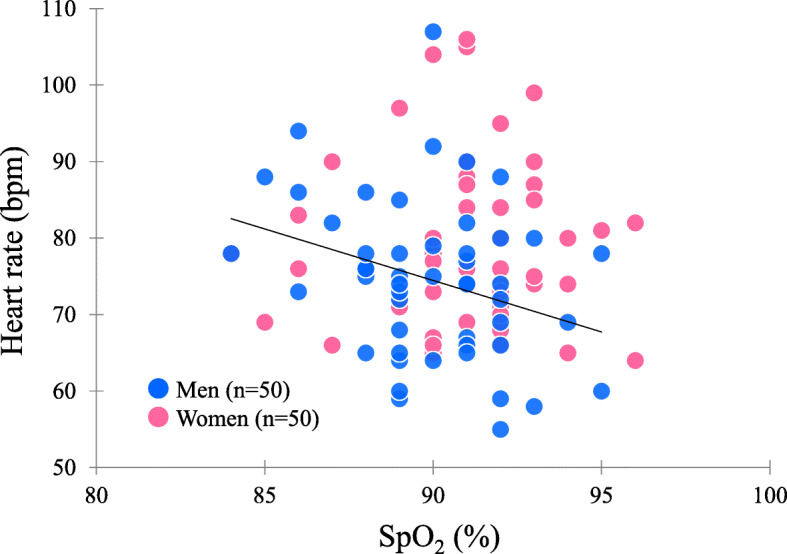
Scatter plot of SpO_2_ and heart rate in Andean highlanders. SpO_2_ is negatively correlated with heart rate (*r* = − 0.307, *p* = 0.030) in men. The solid line indicates the trend for men

In men, SpO_2_ was negatively correlated with heart rate and DBP (Fig. 1, Table 2). In women, SpO_2_ was positively correlated with finger temperature and showed a trend to having a negative correlation with DBP (Table 2).

**Table 2:** Simple correlation coefficients between SpO_2_ and other parameters

	Total (*n* = 100)		Men (*n* = 50)		Women (*n* = 50)	
	*r*	*p* value	*r*	*p* value	*r*	*p* value
Age (years)	− 0.183	0.069	− 0.121	0.401	− 0.179	0.214
BMI (kg/m^2^)	− 0.146	0.148	− 0.161	0.264	− 0.094	0.515
Heart rate (bpm)	− 0.099	0.325	− 0.307	0.030	− 0.013	0.926
SBP (mmHg)	− 0.230	0.021	− 0.160	0.269	− 0.197	0.171
DBP (mmHg)	− 0.261	0.008	− 0.282	0.047	− 0.253	0.076
Hemoglobin (g/dl)	− 0.228	0.023	− 0.085	0.558	− 0.107	0.458
Finger temperature (°C)	0.193	0.055	0.202	0.159	0.341	0.015

*SpO*_*2*_ saturation of percutaneous oxygen, *BMI* body mass index, *SBP* systolic blood pressure, *DBP* diastolic blood pressure

In men, multiple stepwise regression analysis showed that a higher SpO_2_ was significantly correlated with a lower heart rate and a higher finger temperature (Table 3). On the other hand, in women, a higher SpO_2_ was significantly correlated with a higher finger temperature (Table 3). A higher SpO_2_ was related to a higher finger temperature and a lower heart rate in all participants (Table 3).

**Table 3:** Multiple stepwise regression analysis between SpO_2_ and other parameters

	Total (*n* = 100)			Men (*n* = 50)			Women (*n* = 50)		
	*β* (95% CI)	*p* value	*r*^2^	*β* (95% CI)	*p* value	*r*^2^	*β* (95% CI)	*p* value	*r*^2^
Sex (women)	1.446 (0.401, 2.490)	0.007		–	–		–	–	
Finger temperature (°C)	0.286 (0.006, 0.514)	0.014		0.308 (0.032, 0.585)	0.030		0.391 (0.078, 0.704)	0.015	
Heart rate (bpm)	− 0.052 (− 0.099, − 0.006)	0.029		− 0.089 (− 0.151, − 0.256)	0.007		–	–	
Physical activity (yes)	− 0.961 (− 1.944, 0.023)	0.056		–	–		–	–	
DBP (mmHg)	− 0.046 (− 0.106, 0.015)	0.140		–	–		–	–	
			0.210			0.182			0.116

*β* standardized regression coefficient, *95% CI* 95% confidence interval, *DBP* diastolic blood pressure, *r*^*2*^ coefficient of determination for model

Residual analysis showed that individual values were randomly plotted in both sexes (Figs. 3 and 4). There were no significant correlations between the estimated value and the residual in men (*r* = − 0.001, *p* = 0.994) and in women (*r* = − 0.00004, *p* = 0.999). The 95% limits of agreement was − 4.26 to 4.35 in men and − 4.91 to 4.89 in women.

## Discussion

Many previous studies indicated the specific type of high-altitude adaptation of various populations, such as Tibetans, Andeans, and Ethiopians [1, 5, 8–13, 17–19]. On the other hand, studies focusing on variations or sex differences in Andean highlanders have been limited [5, 8]. In the present study, the data for the hemodynamic parameters, focusing primarily on SpO_2_ of young Andean highlanders, are presented.

In the present study, men had significantly lower SpO_2_ than women (Table 1). Although this difference was small and its clinical relevance was unclear, men had higher hemoglobin, which might be enough to deliver the oxygen with lower SpO_2_ compared to women. However, Beall et al. [5] reported that men had higher SpO_2_ than women in Andean people (La Paz, Bolivia). The reason for this inconsistency was not clear, but it might depend on the difference in participants’ smoking habits. Their participants smoked more (62.3% in men and 39.7% in women) [8], and SpO_2_ was overestimated in smokers [20–22]. It is thought that these factors may be related to the inconsistency between the present data and Beall’s data. However, further studies are needed to compare the SpO_2_ between smokers and non-smokers in highlanders.

Hemoglobin and SBP were significantly higher and heart rate was lower in men than women (Table 1). Typically, men have higher hemoglobin and blood pressure and a lower heart rate than women in a sea level environment [23–25], and the present result for hemoglobin is consistent with the previous study of Andean highlanders [8]. These results suggest that the sex differences of hemoglobin, heart rate, and SBP are common in both lowlanders and highlanders.

Men had significantly higher finger temperature than women (Table 1), consistent with previous studies that reported that men had a higher skin temperature and peripheral blood flow in a thermoneutral environment among lowlanders [26, 27].

There was a significant negative correlation between SpO_2_ and heart rate in men, but not in women (Fig. 1, Table 2). In men, multiple regression analysis showed that a lower SpO_2_ was correlated with a higher heart rate after adjusting covariates (Table 3). Although it has been reported that heart rate was decreased after long-term high-altitude exposure [28], the present results indicated that lower SpO_2_ evoked a higher heart rate for higher oxygen delivery. These physiological states were similar to those of lowlanders. The results might depend on sex differences in the sensitivity of the oxygen receptor or the relatively higher heart rate of women compared to men.

**Fig. 2:**
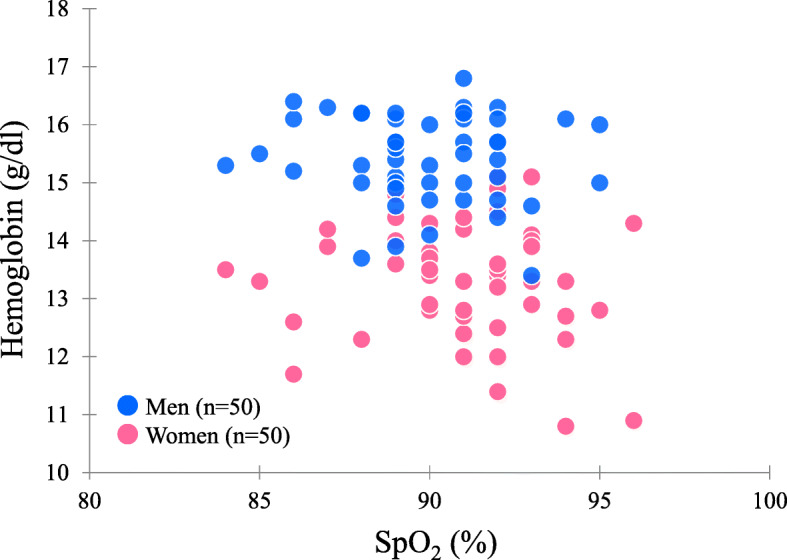
Scatter plot of SpO_2_ and hemoglobin in Andean highlanders.

Beall et al. [8] reported a negative correlation between SpO_2_ and hemoglobin in women and suggested sex differences in hemodynamics in Andean highlanders, whereas SpO_2_ was not correlated with hemoglobin in both sexes in the present study (Fig. 2, Table 2).

In the present study, a lower SpO_2_ was related to or tended to be associated with higher DBP in both sexes (Table 2). Multiple stepwise regression analysis showed that a lower SpO_2_ was significantly correlated to a lower finger temperature in both sexes (Table 3). Finger temperature is important in the evaluation of vasoconstriction. The positive relationship between SpO_2_ and finger temperature probably reflected peripheral circulation levels; an inadequate blood supply to the finger might cause a lower SpO_2_. In the high-altitude environment, vasodilation occurs due to nitric oxide (NO) release from vascular endothelial cells with lower SpO_2_ [29]. Thus, it is expected that a lower SpO_2_ evokes vasodilation and then decreases DBP; previous studies also reported lower blood pressure in highlanders [30]. Therefore, increasing blood flow due to NO release causes increased finger temperature and decreased DBP [30, 31], but this was not consistent with the present study. These responses were also affected by the sex difference in vasoconstrictor responses [32, 33] or sympathetic vasoconstrictor responsiveness via NO mediation [34]. The present results suggest that vasoconstriction (lower finger temperature) was related to a lower SpO_2_, which also causes a higher DBP.

**Fig. 3:**
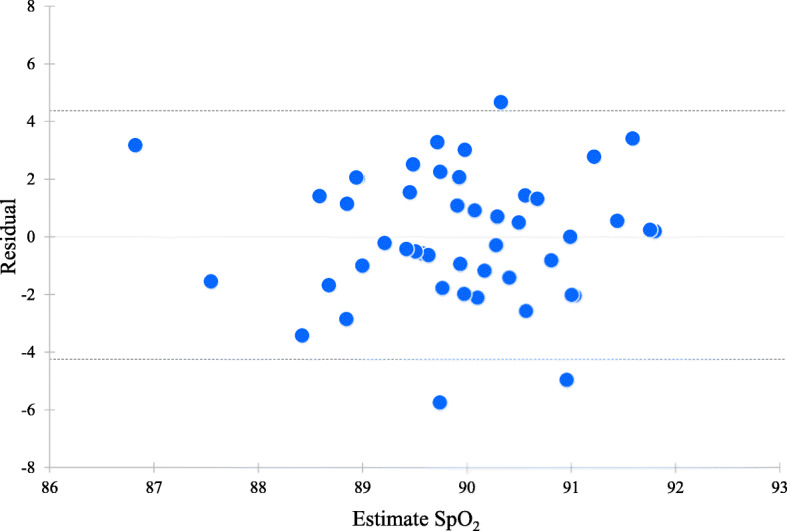
Scatter plot of residual and estimated SpO_2_ values from the regression model in men. The dotted line represents the 95% limits of agreement (− 4.26 to 4.35)

**Fig. 4:**
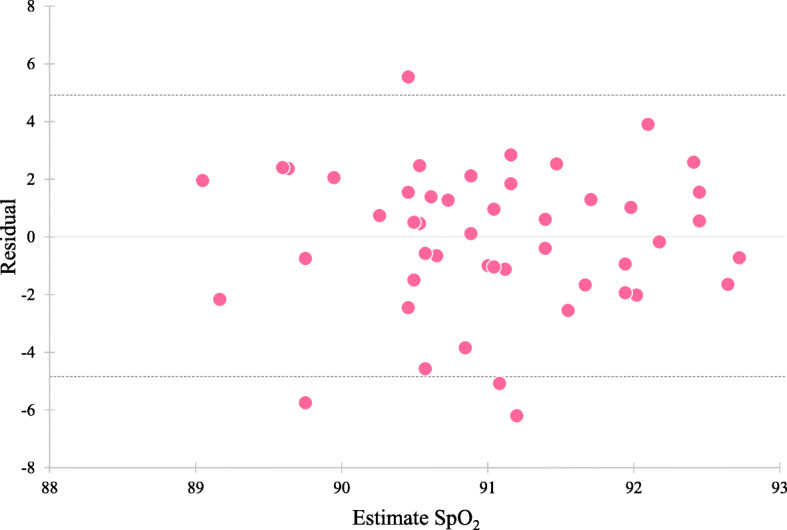
Scatter plot of residual and estimated SpO_2_ values from the regression model in women. The dotted line represents the 95% limits of agreement (− 4.91 to 4.89)

Residual analysis showed that most individual values were randomly plotted within the 95% limits of agreement, and there were no significant correlations between the residual and the estimated SpO_2_ in the regression models (Figs. 3 and 4). Thus, these variations seemed to not be a proportional bias, but random error, such as biological variation. The factors affecting this variation of SpO_2_ were complex. Finger temperature was related to SpO_2_ (Table 3), but a low temperature resulted from reduced perfusion of the limb due to a vasoconstrictor response; it does not mean low arterial oxygen saturation (SaO_2_). A higher SpO_2_ was related to a lower heart rate in all participants (Table 3); therefore, individual variation of SpO_2_ was partly explained by heart rate. This correlation was not significant in women, but it might depend on the sample size. From the above, it was thought that individuals who could have higher SpO_2_ values even in highland areas could live with a lower heart rate, and this might suggest that they are well adapted to the high altitude.

Finally, in the total regression analysis, 21% of the variation in SpO_2_ was explained, but the remaining variation was unclear. Recent studies indicated that the physiological status of highlanders was affected by genetic background, including *EPAS1* and *EGLN1* genes [4, 16, 17, 35–38]. These remaining individual variations might be explained by genetic characteristics or other underlying factors in further studies.

The present study has several limitations. First, the results do not necessarily show a causal relationship because this study was cross-sectional in design. Second, information on other determinants (e.g., ventilation, nutritional status, or menstrual cycle) contributing to SpO_2_ was not clear. Third, hemoglobin concentration was an estimated value that was difficult to compare to values reported by other studies. Finally, the participants were university students and may differ from the general population.

## Conclusion

In conclusion, the present study showed that SpO_2_ values of Andean highlanders were not homogeneous. Residual analysis showed that the plots of SpO_2_ were random. In men, a higher SpO_2_ was related to a lower heart rate and finger temperature. A higher SpO_2_ was related to a higher finger temperature in women. These results suggest that there are individual variations and sex differences in the hemodynamics of high-altitude adaptation in Andean highlanders.

## Data Availability

Not applicable.

## Abbreviations

95% CI: 95% confidence interval
AMS: Acute mountain sickness
BMI: Body mass index
DBP: Diastolic blood pressure
EGLN1: Egl-9 family hypoxia-inducible factor 1
EPAS1: Endothelial PAS domain-containing protein 1
Hb: Hemoglobin
HbA1c: Glycosylated hemoglobin
HIF: Hypoxia-inducible factor
NO: Nitric oxide
SaO_2_: Arterial oxygen saturation
SBP: Systolic blood pressure
SpO_2_: Percutaneous arterial oxygen saturation
